# Distinct histological alterations of cortical interneuron types in mouse models of Huntington’s disease

**DOI:** 10.3389/fnins.2022.1022251

**Published:** 2022-09-26

**Authors:** Kerstin Voelkl, Elena Katharina Schulz-Trieglaff, Rüdiger Klein, Irina Dudanova

**Affiliations:** ^1^Department of Molecules–Signaling–Development, Max Planck Institute for Biological Intelligence, Martinsried, Germany; ^2^Molecular Neurodegeneration Group, Max Planck Institute for Biological Intelligence, Martinsried, Germany; ^3^Center for Anatomy, Faculty of Medicine and University Hospital Cologne, University of Cologne, Cologne, Germany

**Keywords:** Huntington’s disease, R6/2 mouse model, zQ175DN mouse model, cerebral cortex, GABAergic interneurons, immunostaining, genetic tracing, mHTT inclusion bodies

## Abstract

Huntington’s disease (HD) is a debilitating hereditary motor disorder caused by an expansion of the CAG triplet repeat in the Huntingtin gene. HD causes neurodegeneration particularly in the basal ganglia and neocortex. In the cortex, glutamatergic pyramidal neurons are known to be severely affected by the disease, but the involvement of GABAergic interneurons remains unclear. Here, we use a combination of immunostaining and genetic tracing to investigate histological changes in three major cortical interneuron types — parvalbumin (PV), somatostatin (SST), and vasoactive intestinal peptide (VIP) interneurons — in the R6/2 and zQ175DN mouse models of HD. In R6/2 mice, we find a selective reduction in SST and VIP, but not PV-positive cells. However, genetic labeling reveals unchanged cell numbers for all the interneuron types, pointing to molecular marker loss in the absence of cell death. We also observe a reduction in cell body size for all three interneuron populations. Furthermore, we demonstrate progressive accumulation of mutant Huntingtin (mHTT) inclusion bodies in interneurons, which occurs faster in SST and VIP compared to PV cells. In contrast to the R6/2 model, heterozygous zQ175DN knock-in HD mice do not show any significant histological changes in cortical cell types at the age of 12 months, apart from the presence of mHTT inclusions, which are abundant in pyramidal neurons and rare in interneurons. Taken together, our findings point to differential molecular changes in cortical interneuron types of HD mice.

## Introduction

Huntington’s disease (HD) is a hereditary movement disorder that typically starts in midlife and is inevitably lethal within 10–20 years after onset. The disease manifests with a triad of clinical signs, including uncontrollable movements (chorea) later replaced by akinesia, psychiatric symptoms such as depression, and cognitive impairment culminating in dementia (Tabrizi et al., 2020). The cause of HD is a pathological CAG trinucleotide repeat expansion in the first exon of the Huntingtin gene (The Huntington’s Disease Collaborative Research Group, 1993), which leads to an elongated polyglutamine (polyQ) tract in the mutant Huntingtin (mHTT) protein. mHTT is aggregation-prone and forms inclusion bodies (IBs), typically localized in neuronal nuclei and in the neurites (DiFiglia et al., 1997). HD causes severe neurodegeneration, which is most prominent in the striatum and the neocortex (Vonsattel and DiFiglia, 1998; Waldvogel et al., 2015).

The neocortex consists of ∼80% glutamatergic pyramidal neurons (also called principal cells, PCs) and ∼20% GABAergic interneurons (DeFelipe, 2002). PCs are clearly vulnerable to HD and their numbers are markedly reduced in postmortem brain tissue from patients (Cudkowicz and Kowall, 1990; Macdonald and Halliday, 2002; Thu et al., 2010; Estrada-Sanchez and Rebec, 2013). In contrast, the involvement of the cortical GABAergic interneurons in HD pathology has for a long time remained unclear (Blumenstock and Dudanova, 2020). Cortical interneurons are very diverse in terms of morphology, electrophysiological properties, function, and molecular profiles (Tremblay et al., 2016). They can be subdivided into three major populations based on the expression of characteristic molecular markers: parvalbumin (PV)-positive, somatostatin (SST)-positive, and 5HT3a-receptor-positive cells (Xu et al., 2010). Within the heterogeneous population of 5HT3a-receptor-positive cells, a major subclass expresses the marker vasoactive intestinal peptide (VIP). A number of additional interneuron markers such as calretinin and calbindin label parts of the major three interneuron populations described above (Gonchar et al., 2007; Xu et al., 2010).

Early histological studies of postmortem brains from HD patients found no changes in the number, distribution or morphology of different interneuron types (Cudkowicz and Kowall, 1990; Macdonald and Halliday, 2002). However, more recent reports described reductions in specific interneuron populations in various cortical areas (Kim et al., 2014; Mehrabi et al., 2016). Interestingly, cell loss in HD cortex appears to correlate with the disease symptomatology. In particular, HD patients with predominantly motor symptoms exhibit loss of PCs and calbindin-positive, but not PV or calretinin-positive interneurons in the primary motor cortex. In contrast, patients with mood disorder show changes specifically in the anterior cingulate cortex, where all studied populations (PCs as well as PV, calbindin, and calretinin interneurons) are reduced (Thu et al., 2010; Kim et al., 2014). Furthermore, studies in genetic mouse models provided evidence for a contribution of cortical interneuron dysfunction to disease mechanisms (Gu et al., 2005; Spampanato et al., 2008; Cummings et al., 2009; Dougherty et al., 2014). While some of these studies focused on PV cells in particular (Spampanato et al., 2008; Dougherty et al., 2014), the SST and VIP populations have not yet been directly examined in the context of HD.

Here, we have performed a thorough morphological analysis of three major populations of cortical interneurons – PV, SST, and VIP cells – in two widely used mouse models of HD, the R6/2 transgenic fragment model (Mangiarini et al., 1996) and the zQ175DN full-length knock-in model (Menalled et al., 2012; Southwell et al., 2016). While both mouse models exhibit mHTT inclusion bodies in interneurons, R6/2 mice additionally show interneuron type-specific, age-dependent changes in molecular marker expression and cell body size.

## Results

### Selective decrease in somatostatin and vasoactive intestinal peptide-positive interneurons in R6/2 mice

To start investigating the impact of HD on cortical interneurons, we performed immunostainings for the molecular markers of the three major interneuron populations in the primary motor cortex of R6/2 mice and wildtype littermates. R6/2 transgenic mice express an N-terminal fragment of mHTT-exon1 under the human *HTT* promoter (Mangiarini et al., 1996). The line is characterized by a fast disease progression and a short life span of 3–5 months. The length of the CAG tract in our colony amounted to 201 ± 10 repeats.

**FIGURE 1:**
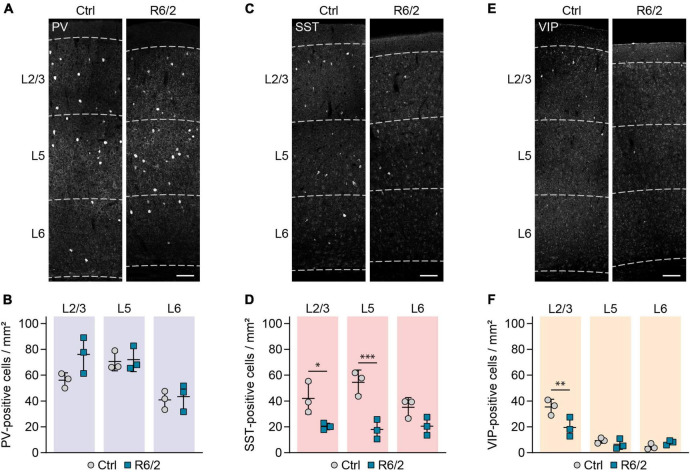
Selective reduction in SST and VIP-positive interneurons in the primary motor cortex of R6/2 mice. **(A,C,E)** Representative images of the primary motor cortex immunostained for the indicated molecular markers. Cortical layers are marked with dashed lines and labeled on the left. **(B,D,F)** Quantifications of cell densities per layer. *N* = 3 mice for all the groups. RM two-way ANOVA with Bonferroni’s multiple comparisons test. PV cells: Genotype, n.s.; Layer, ^***^p = 0.0001; Genotype × layer, n.s. SST cells: Genotype, ^**^p = 0.005; Layer, n.s.; Genotype × Layer, n.s. VIP: Genotype, n.s.; Layer, ^*⁣*⁣**^p < 0.0001; Genotype × layer, ^**^p = 0.0066. Significant pairwise comparisons are indicated on the graphs. *p < 0.05; ^**^p < 0.01; ^***^p < 0.001. Scale bars in panels **(A,C,E)** are 100 μm.

We quantified the density of PV-immunopositive (PV+), SST+, and VIP+ cell bodies in cortical layer 2/3, layer 5 and layer 6 at the age of 12 weeks, corresponding to an advanced disease stage (Mangiarini et al., 1996; Carter et al., 1999). In agreement with our previous findings in R6/2 mice and HD patients (Burgold et al., 2019), we did not detect any loss of PV+ cells (Figures 1A,B). In contrast, there was a significant reduction of SST+ neurons in layer 2/3 and layer 5 (Figures 1C,D). The density of VIP+ cells was also significantly reduced in layer 2/3, where the majority of these cells are located (Figures 1E,F).

**FIGURE 2:**
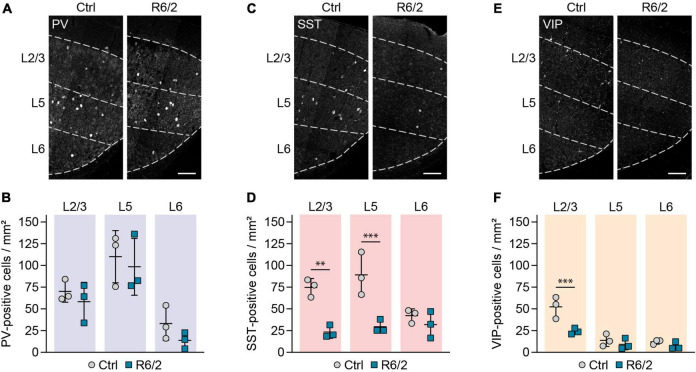
Selective reduction in SST and VIP-positive interneurons in the cingulate cortex of R6/2 mice. **(A,C,E)** Representative images of the cingulate cortex immunostained for the indicated molecular markers. Cortical layers are marked with dashed lines and labeled on the left. **(B,D,F)** Quantifications of cell densities per layer. *N* = 3 mice for all the groups. RM two-way ANOVA with Bonferroni’s multiple comparisons test. PV cells: Genotype, n.s.; Layer, ^***^p = 0.0003; Genotype × layer, n.s. SST cells: Genotype, ^**^p = 0.0034; Layer, n.s.; Genotype × layer, *p = 0.0285. VIP cells: Genotype, *p = 0.0272; Layer, ^*⁣*⁣**^p < 0.0001; Genotype × layer, *p = 0.0176. Significant pairwise comparisons are indicated on the graphs. ^**^p < 0.01; ^***^p < 0.001. Scale bars in panels **(A,C,E)** are 100 μm.

As histological analyses of human postmortem tissue revealed loss of interneurons in the anterior cingulate cortex of HD patients with mood disorder (Kim et al., 2014), we next quantified interneuron densities in the anterior cingulate cortex of R6/2 mice. Changes in forced swim test have been previously reported in this mouse model, suggestive of depressive-like behaviors (Ciamei et al., 2015). Our findings in the anterior cingulate cortex were overall similar to the primary motor cortex, with unchanged density of PV+ cells and reduced density of SST+ and VIP+ cells (Figures 2A–F). Taken together, these results demonstrate a selective decrease in SST and VIP-immunopositive cells in different cortical areas of R6/2 mice.

### Decrease in somatostatin and vasoactive intestinal peptide markers in R6/2 mice is not due to cell loss

**FIGURE 3:**
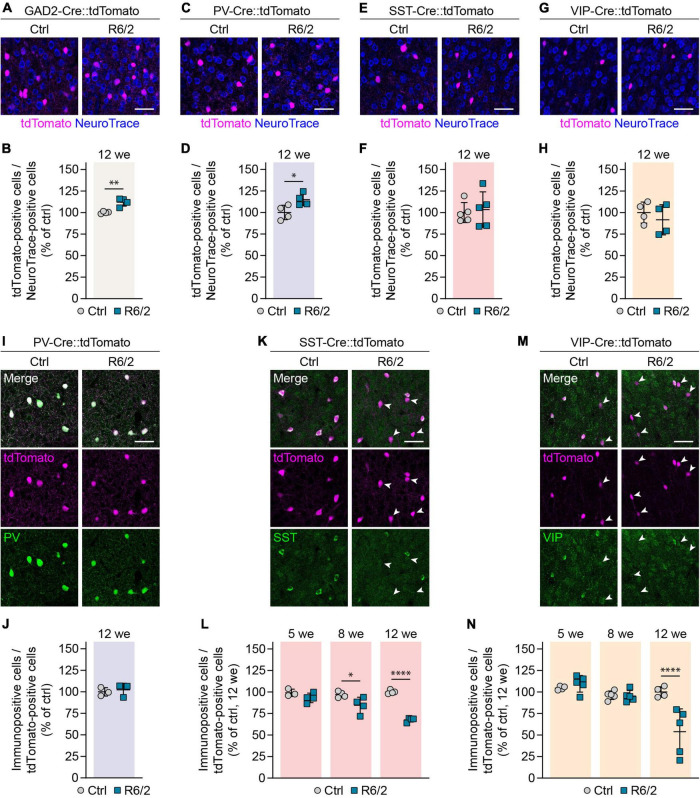
Genetic labeling reveals absence of cortical interneuron cell loss in R6/2 mice. **(A,C,E,G)** Representative images of the primary motor cortex layer 2/3 from 12-week-old mice with the indicated interneuron populations labeled with tdTomato. The sections were stained with NeuroTrace. **(B,D,F,H)** Quantifications of the genetically labeled cells. Values were normalized to controls. *N* = 4–5 mice. Unpaired two-sided *t*-test. **(I,K,M)** Representative images of the primary motor cortex layer 2/3 from 12-week-old mice. Genetic tracing was combined with immunostaining for the indicated markers. Arrowheads in panels **(K,M)** point to tdTomato-labeled cells showing no expression of the respective molecular marker. **(J,L,N)** Quantifications of the fraction of genetically labeled cells immunopositive for the respective marker. Values were normalized to 12-week-old controls. PV: *N* = 3 mice. Unpaired two-sided *t*-test, n.s. SST: *N* = 3–4 mice. Two-way ANOVA with Bonferroni’s multiple comparisons test. ANOVA: Genotype, ^*⁣*⁣**^p < 0.0001; Age, ^**^p = 0.0012; Genotype × age, ^***^p = 0.0004. Significant pairwise multiple comparisons are indicated on the graph. VIP: *N* = 4–5 mice. Two-way ANOVA with Bonferroni’s multiple comparisons test. ANOVA: Genotype, ^**^p = 0.0083; Age, ^***^p = 0.0002; Genotype × age, ^***^p = 0.0005. Significant pairwise multiple comparisons are indicated on the graph. *p < 0.05; ^**^p < 0.01; ^*⁣*⁣**^p < 0.0001. Scale bars in panels **(A,C,E,G,I,K,M)** are 50 μm.

The reduction in interneuron markers observed in R6/2 mice raised the possibility that GABAergic interneurons undergo cell death in this HD model. To differentiate between true cell loss and decreased expression of the respective molecular markers, we genetically labeled GABAergic interneurons by crossing R6/2 mice to the GAD2-Cre line specific to GABAergic cells (Taniguchi et al., 2011) and to the Cre-dependent Ai9 Rosa26-LSL-tdTomato reporter (Madisen et al., 2010). We then stained brain sections with NeuroTrace to label all neurons. In agreement with previous reports (Stack et al., 2005; Cepeda-Prado et al., 2012; Rattray et al., 2013), cortex width was reduced by 11% in 12-week-old R6/2 mice, while overall neuronal density was increased by 14% (Supplementary Figures 1A–C). The increased density of neurons is likely due to tissue shrinkage in the absence of cell loss, as suggested previously (Rattray et al., 2013). We then quantified the number of tdTomato+ GABAergic interneurons, and normalized it to the total number of neurons detected with NeuroTrace to account for the altered cell density in R6/2 mice. We observed a 12% increase in the density of GABAergic interneurons (Figures 3A,B), arguing against an overall reduction of interneuron numbers.

To analyze cell numbers for the specific interneuron subtypes, we crossed R6/2 mice to tdTomato mice and to PV-Cre (Hippenmeyer et al., 2005), SST-Cre and VIP-Cre lines (Taniguchi et al., 2011), which were reported to faithfully label the respective interneuron populations with minimal overlap between each other (Pfeffer et al., 2013). We did not detect significant reductions in any of the populations (Figures 3C–H), suggesting that the decrease in both SST+ and VIP+ interneurons is not due to a loss of cell bodies, but rather to a loss of the respective molecular marker in a fraction of cells. To directly test this possibility, we combined genetic labeling of the three interneuron populations with immunostaining for the respective molecular markers. For SST and VIP populations, we also performed these experiments at earlier ages (5 and 8 weeks in addition to 12 weeks) in order to distinguish whether the reduction in interneuron markers was due to a developmental defect, or a result of an adult-onset degenerative process. As expected, immunopositive and genetically labeled cells overlapped well for all three populations in control mice, and for the PV population in R6/2 mice (Figures 3I–N). For SST and VIP cells in R6/2 animals, the overlap was initially similar to control mice, but the fraction of immunopositive cells decreased with disease progression. For SST neurons, we observed a gradual decline after 5 weeks of age (Figure 3L), while for VIP cells, the change occurred more abruptly between 8 and 12 weeks (Figure 3N). These results point to a selective loss of molecular markers specifically in SST and VIP, but not PV interneurons, in the absence of cell loss.

### Unaltered numbers of cortical interneurons in zQ175DN mice

**FIGURE 4:**
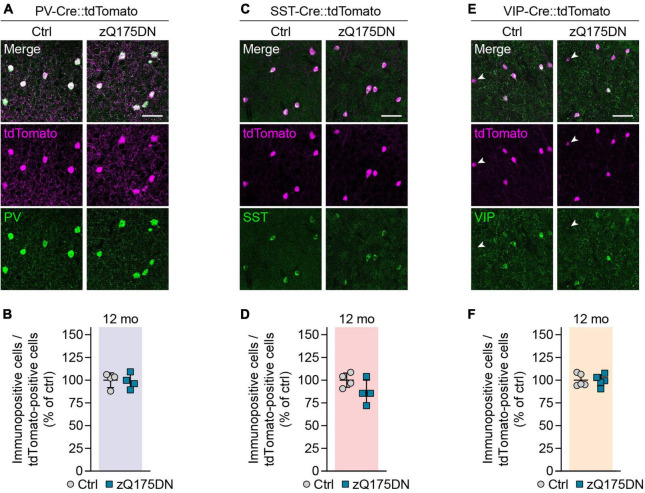
Genetic labeling of interneurons in zQ175DN mice. **(A,C,E)** Representative images of the primary motor cortex layer 2/3 from 12-month-old zQ175DN mice. Genetic tracing was combined with immunostaining for the indicated markers. Arrowheads in panel **(E)** point to tdTomato-labeled cells showing no VIP staining. **(B,D,F)** Quantifications of the fraction of genetically labeled cells immunopositive for the respective marker. Values were normalized to controls. *N* = 4–5 mice. Unpaired two-sided *t*-test. No significant differences were observed. Scale bars in panels **(A,C,E)** are 50 μm.

We next investigated cortical interneurons in zQ175DN mice, a full-length knock-in model of HD that expresses mHTT from the endogenous murine *Htt* locus (Menalled et al., 2012; Southwell et al., 2016). The CAG repeat number in our colony amounted to 203 ± 12 repeats and was similar to that in R6/2 mice. Heterozygous zQ175DN animals were analyzed at 12 months, an age when they were reported to display mild defects in motor behavior and corticostriatal synaptic transmission (Heikkinen et al., 2012; Southwell et al., 2016). Although slight cortical atrophy has been previously observed in this mouse model (Heikkinen et al., 2012; Southwell et al., 2016), we did not find any change in the cortex width or overall neuron density at 12 months (Supplementary Figures 1D–F). Genetic labeling of PV, SST, and VIP populations combined with immunostaining for the respective molecular markers also did not reveal any significant cell loss or marker loss (Figures 4A–F). In summary, no changes were observed in the numbers or molecular marker expression in the three major cortical interneuron types of heterozygous zQ175DN mice.

### Reduced cell body size in cortical populations of R6/2, but not zQ175DN mice

**FIGURE 5:**
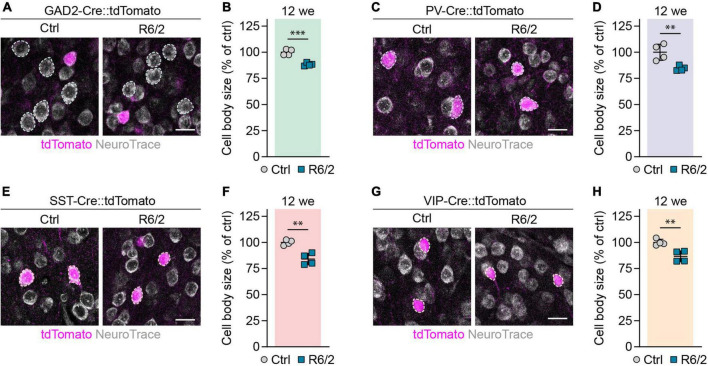
Reduction in cell body size of cortical neuron populations in R6/2 mice. **(A,C,E,G)** Representative images of the primary motor cortex layer 2/3 from 12-week-old mice with the indicated populations labeled with tdTomato. The sections were stained with NeuroTrace. Dashed lines mark cells of the respective analyzed populations. **(B,D,F,H)** Quantifications of cell body area, normalized to littermate control mice. *N* = 4. Unpaired two-sided *t*-test. ^***^p < 0.01; ^***^p < 0.001. Scale bars in panels **(A,C,E,G)** are 20 μm.

**FIGURE 6:**
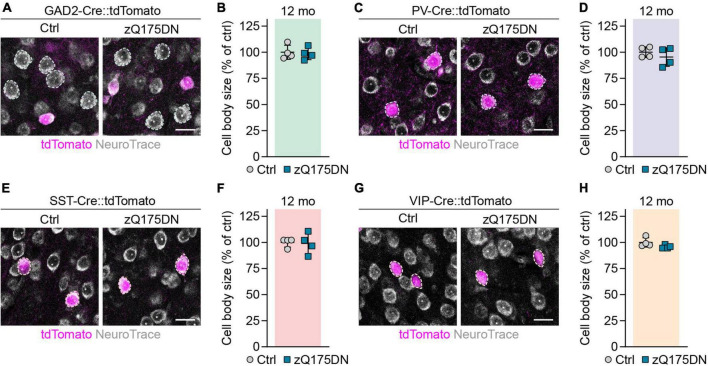
Analysis of cell body size in cortical neuron populations of zQ175DN mice. **(A,C,E,G)** Representative images of the primary motor cortex layer 2/3 from 12-month-old mice with the indicated populations labeled with tdTomato. The sections were stained with NeuroTrace. Dashed lines mark cells of the respective analyzed populations. **(B,D,F,H)** Quantifications of cell body area, normalized to littermate controls. *N* = 4. Unpaired two-sided *t*-test. No significant differences were observed. Scale bars in panels **(A,C,E,G)** are 20 μm.

Shrinkage of neuronal cell bodies is known to occur in HD patients (Thu et al., 2010), but no cortical cell type-specific data has been reported for HD mouse models so far. We measured the area of cell bodies of PCs and different interneurons in the primary motor cortex of 12-week-old R6/2 and 12-month-old zQ175DN mice. PCs were detected as tdTomato-negative neurons in GAD2-Cre::tdTomato mice, while PV, SST, and VIP interneurons were genetically labeled with the respective specific Cre lines. We found a 12% decrease in cell body size of PCs in R6/2 mice compared to control littermates (Figures 5A,B). Cell body size of PV, SST, and VIP interneurons was also reduced by 15, 16, and 13%, respectively (Figures 5C–H). In contrast, we did not detect any significant changes of cell body area in any of the studied populations of zQ175DN mice (Figures 6A–H). Taken together, these analyses reveal a similar degree of cell shrinkage in glutamatergic PCs and GABAergic interneurons of R6/2 mice, while no changes in neuronal soma size are evident in the cortex of zQ175DN mice.

### Loss of perisomatic synaptic terminals of parvalbumin interneurons in R6/2 mice

**FIGURE 7:**
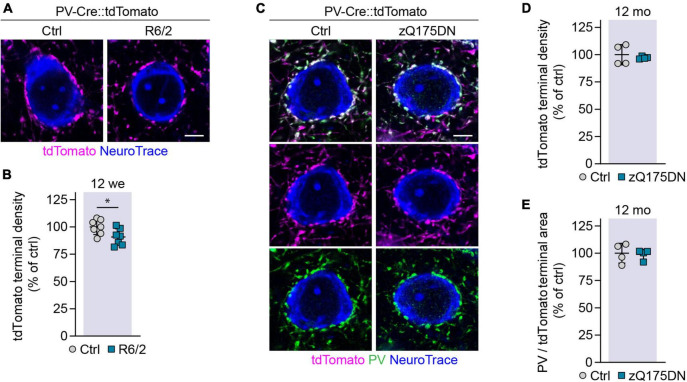
Analysis of PV synaptic terminals on PCs in R6/2 and zQ175 mice. **(A)** Representative images of PV synaptic terminals genetically labeled with tdTomato, surrounding NeuroTrace-stained PCs in 12-week-old R6/2 mice and littermate controls. **(B)** Quantification of PV terminals on layer 2/3 and layer 5 PCs. Values were normalized to controls. *N* = 7 mice. Unpaired two-sided *t*-test. **(C)** Representative images of PV synaptic terminals genetically labeled with tdTomato and immunostained for PV, surrounding NeuroTrace-stained PCs in 12-month-old zQ175DN mice and littermate controls. **(D)** Quantification of the density of genetically labeled PV terminals on layer 2/3 and layer 5 PCs. Values were normalized to controls. *N* = 4 mice. Unpaired two-sided *t*-test. No significant differences were found. **(E)** Ratio of the PV immunostaining area and tdTomato labeling area around layer 2/3 and layer 5 PCs in zQ175DN and control mice. Values were normalized to controls. *N* = 4 mice. Unpaired two-sided *t*-test. No significant differences were observed. *p < 0.05. Scale bars in panels **(A,C)** are 5 μm.

We have previously found a reduction in perisomatic PV synapses on cortical PCs in R6/2 mice and human HD cases (Burgold et al., 2019). As these findings were based on immunostaining, the question remained whether the change in PV synapse staining was due to synapse loss or to a reduced expression of the marker. We therefore took advantage of the genetic labeling in PV-Cre::tdTomato mice and quantified the density of tdTomato-positive synaptic terminals surrounding PC cell bodies in layer 2/3 and layer 5. PV interneurons are known to form synapses at or very close to the soma, and to target exclusively PV cells and PCs (Tremblay et al., 2016). PV synaptic endings on PCs can therefore be recognized in PV-Cre:tdTomato mice as tdTomato-labeled puncta surrounding unlabeled cell bodies. We observed a small, but significant reduction in the density of PV synapses surrounding PCs in R6/2 mice (Figures 7A,B). This argues for a true loss of perisomatic GABAergic PV synapses, although we cannot exclude the possibility that PV expression at the synapse might also be reduced. In contrast, we did not detect any changes in PV terminals in 12-month-old heterozygous zQ175DN mice either by immunostaining or by genetic labeling of PV cells (Figures 7C–E).

### Mutant Huntingtin inclusion body load in cortical cell types of Huntington’s disease mice

**FIGURE 8:**
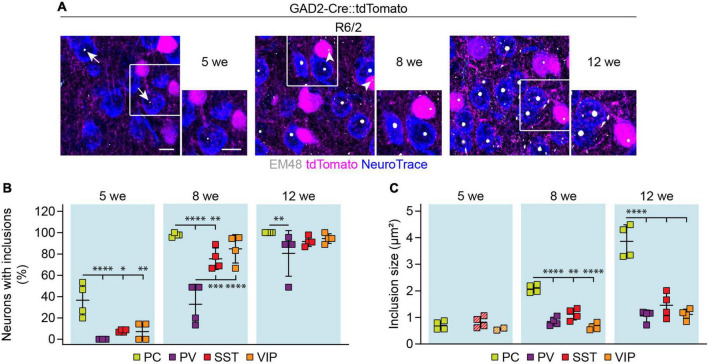
mHTT inclusion body load in cortical cell types of R6/2 mice. **(A)** Representative images of the primary motor cortex layer 2/3 from R6/2 mice at the indicated ages. GABAergic cells were genetically labeled with tdTomato, all neurons were stained with NeuroTrace, mHTT inclusion bodies were detected with the EM48 antibody. Insets on the right show higher magnification of the areas indicated by the boxes. Arrows point to mHTT inclusion bodies in PCs, arrowheads indicate inclusions in interneurons. **(B)** Fraction of cells with mHTT inclusions. *N* = 3–4 mice. Two-way ANOVA with Bonferroni’s multiple comparisons test following arcsin transformation. ANOVA: Cell type, ^*⁣*⁣**^p < 0.0001; Age, ^*⁣*⁣**^p < 0.0001; Cell type × age, n.s. Significant pairwise multiple comparisons are indicated on the graph. **(C)** Inclusion area. Quantification was only performed in the mice that showed inclusions in the respective cell type. Striped data points indicate animals where < 20% of respective neurons had inclusions. *N* = 2–4 mice. Two-way ANOVA with Bonferroni’s multiple comparisons test (only 8-week and 12-week time points were included in the statistical analysis). ANOVA: Cell type, ^*⁣*⁣**^p < 0.0001; Age, ^*⁣*⁣**^p < 0.0001; Cell type × age, ^***^p = 0.0001. Significant pairwise multiple comparisons are indicated on the graph. *p < 0.05; ^**^p < 0.01; ^***^p < 0.001; ^*⁣*⁣**^p < 0.0001. Scale bars in panel **(A)** are 10 μm.

We next asked whether cortical interneuron types display differences in the load of mHTT inclusion bodies. Cortical sections from HD mice with tdTomato-labeled PV, SST, or VIP cells were stained with the EM48 antibody (Figure 8A), which labels mHTT aggregates (Gutekunst et al., 1999). To quantify inclusion bodies in glutamatergic PCs, we used genetic tracing in GAD2-Cre::tdTomato mice and analyzed tdTomato-negative neurons. The fraction of neurons bearing mHTT inclusions as well as the area of individual inclusion bodies were analyzed at 5, 8, and 12 weeks of age in R6/2 mice and at 12 months of age in zQ175DN mice.

In R6/2 mice of all age groups, inclusion bodies were more frequent in PCs than in interneurons (Figures 8A,B), consistent with previous reports (Meade et al., 2002). The size of inclusions was also larger in PCs than in interneurons starting from 8 weeks (Figure 8C). Comparisons between interneuron types revealed slower inclusion formation in PV cells, which was the only population without inclusions at 5 weeks, and displayed significantly less inclusions than SST and VIP cells at 8 weeks (Figure 8B). We found no significant differences in inclusion size between the interneuron types (Figure 8C).

**FIGURE 9:**
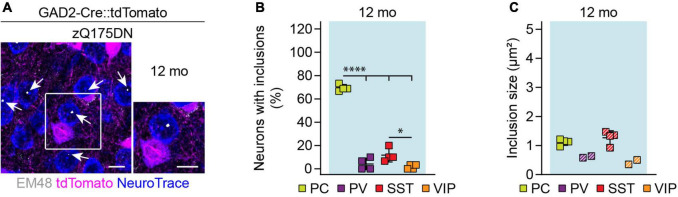
mHTT inclusion body load in cortical cell types of zQ175DN mice. **(A)** Representative images of the primary motor cortex layer 2/3 from 12-month-old zQ175DN mice. GABAergic cells were genetically labeled with tdTomato, all neurons were stained with NeuroTrace, mHTT inclusion bodies were detected with the EM48 antibody. Inset shows a higher magnification image of the area indicated by the box. Arrows point to mHTT inclusion bodies in PCs. **(B)** Fraction of cells with mHTT inclusions. *N* = 4 mice. One-way ANOVA with Bonferroni’s multiple comparisons test following arcsin transformation. ANOVA: ^*⁣*⁣**^p < 0.0001. Significant pairwise multiple comparisons are indicated on the graph. **(C)** Inclusion area. Quantification was only performed in the mice that showed inclusions in the respective cell type. Striped data points indicate animals where < 20% of respective neurons had inclusions. *N* = 2–4 mice. No statistical analysis was performed because of the low numbers of inclusions in cortical interneurons. *p < 0.05; ^*⁣*⁣**^p < 0.0001. Scale bars in panel **(A)** are 10 μm.

Similar to R6/2 mice, zQ175DN mice showed a significantly higher frequency of mHTT inclusions in PCs compared to interneurons (Figures 9A,B). Among interneurons, the greatest frequency of inclusions was observed in SST cells. Inclusion size was comparable between neuronal cell types at this age (Figure 9C). Taken together, these results point to a higher mHTT inclusion load in glutamatergic than GABAergic neurons in both R6/2 and zQ175DN mice.

## Discussion

Here we have histologically examined the effects of the Huntingtin mutation on cortical interneurons and uncovered distinct changes in the three major interneuron cell types in the early-onset R6/2 model of HD. While all three interneuron types exhibited unaltered cell numbers and smaller cell bodies, there was a selective loss of characteristic molecular markers in SST and VIP, but not PV cells. SST and VIP cells also developed mHTT inclusions faster than PV cells. Although PV cells thus seem less affected, we did observe a loss of their synaptic terminals on PC somata, suggesting that mHTT has an impact on all the examined interneuron populations.

Importantly, our findings in R6/2 mice are overall in agreement with studies in postmortem brain tissue from HD patients, where differential alterations have also been found in cortical interneuron types. For example, we have previously shown that perisomatic PV terminals on PCs are reduced, while PV cell numbers are not changed in the primary motor cortex of HD cases (Burgold et al., 2019). Although SST and VIP interneurons have not yet been directly examined in human HD, a reduction in calbindin-positive cells has been described in the motor cortex of patients with motor symptomatology (Kim et al., 2014), and calbindin cells show a considerable overlap with the SST population (Gonchar et al., 2007). In addition to the cerebral cortex, differential changes in interneuron types were also reported in the striatum of HD patients. Here, PV cells are selectively lost, whereas other interneurons are largely spared, including neuropeptide Y/SST/nitric oxide synthase-expressing and calretinin-expressing GABAergic interneurons, as well as cholinergic interneurons (Reiner et al., 2013; Reiner and Deng, 2018).

None of the morphological alterations of cortical interneurons found in R6/2 mice were observed in zQ175DN mice at the age of 12 months. Of note, we also found that mHTT inclusion bodies developed very slowly in this model, so that the frequency and size of inclusions in 12-month-old zQ175DN mice (Figures 9B,C) were similar to those in 5-week-old R6/2 mice (Figures 8B,C). These results are consistent with the R6/2 model having overall severe phenotypes and early lethality (Mangiarini et al., 1996; Carter et al., 1999), whereas the zQ175DN model shows slower disease progression, mild phenotypes and a normal life span (Heikkinen et al., 2012; Southwell et al., 2016; Zeitler et al., 2019). It is therefore possible that similar morphological and molecular defects as observed in R6/2 mice might still appear in cortical interneurons of zQ175DN at a later age than examined in this study.

Our results call for caution in analyzing cell loss based only on immunostaining for a specific cell marker, as our genetic tracings in mice show that reductions in marker expression can occur without cell loss. It is therefore not clear to what extent the decreased numbers of certain interneurons previously observed in human postmortem tissue from HD patients (Kim et al., 2014; Mehrabi et al., 2016) are due to cell death rather than altered expression of the respective marker proteins. Indeed, single-nucleus RNA-sequencing (snRNA-seq) studies in HD mice available to date have demonstrated pronounced downregulation of specific cell-type markers in striatal cell types, presumably reflecting impaired maintenance of cell identity (Lee et al., 2020; Malaiya et al., 2021). We propose that similar processes could also occur in cortical interneurons. In agreement with our data, snRNA-seq profiling of the cingulate cortex in HD patients revealed downregulation of SST and VIP transcripts in neuronal cell clusters, although the proportion of neuronal nuclei among the total pool of nuclei was also reduced in this study (Al-Dalahmah et al., 2020). More detailed single-cell transcriptomic analyses of human tissue will help resolve this question.

How *HTT* mutation causes molecular marker loss in certain neuronal populations and the physiological significance of these changes remain to be explored. Interestingly, both SST and VIP are neuropeptides with important roles in the nervous system. They can be released at synapses, and are known to modulate neuronal activity and cognitive functions (Liguz-Lecznar et al., 2016; Cunha-Reis et al., 2021; Song et al., 2021). Decreased expression of SST has been documented in a number of brain disorders including Alzheimer’s disease (AD), Parkinson’s disease, depression and schizophrenia. In AD, SST deficiency is believed to contribute to memory loss, and restoration of SST levels improves cognition and other disease phenotypes (Liguz-Lecznar et al., 2016; Song et al., 2021). VIP has anti-inflammatory, neurogenic and neuroprotective effects (Cunha-Reis et al., 2021). Its expression is also reduced in AD brains, and overexpression of VIP is beneficial in AD mouse models (Offen et al., 2000; Passemard et al., 2011; Song et al., 2012). Further research is needed to investigate the functional consequences of the loss of these peptides in the cortex, and to explore the potential of SST- and VIP-related drugs in the context of HD.

Taken together, our findings support the notion that mHTT, although broadly expressed, causes differential changes in neuronal cell types. Elucidation of the mechanisms underlying this selectivity is an exciting matter for future investigations.

## Materials and methods

### Mice

All animal experiments were approved by the Government of Upper Bavaria (animal protocols 55.2-1-54-2532-168-14 and ROB-55.2-2532.Vet_02-20-5) and conducted in accordance with the relevant guidelines and regulations. Mice were housed in the institutional animal facility under controlled environment (22 ± 1°C, 55 ± 5% humidity, 14 h/10 h light/dark cycle) with free access to food and water. The R6/2 line (Mangiarini et al., 1996; JAX stock #002810) was maintained by breeding transgenic males with F1 hybrid females derived from crossing CBA (Janvier Labs) with C57BL/6 (Janvier Labs) mice. zQ175DN (Menalled et al., 2012; Southwell et al., 2016; JAX stock #029928), tdTomato (Madisen et al., 2010; JAX stock #007909), GAD2-Cre (Taniguchi et al., 2011; JAX stock #010802), PV-Cre (Hippenmeyer et al., 2005; JAX stock #008069), SST-Cre (Taniguchi et al., 2011; JAX stock #018973), and VIP-Cre (Taniguchi et al., 2011; JAX stock #031628) mice were kept on C57BL/6 background. For genetic tracing experiments, hemizygous R6/2 males or heterozygous zQ175DN mice were crossed to mice heterozygous for the Cre allele and homozygous for the tdTomato reporter allele. Immunostainings shown in Figures 1, 2 were performed on female mice. In all other experiments, groups of mixed sex were used. CAG repeat length was determined by Laragen, Inc., and amounted to 201 ± 10 and 203 ± 12 (SEQ CAG No., mean ± SD) for R6/2 and zQ175DN mice, respectively. No effect of CAG repeat length was observed in any of the experiments.

### Immunostaining and image analysis

Mice were transcardially perfused with phosphate-buffered saline (PBS) for 4 min followed by 4% paraformaldehyde (PFA) in PBS for 6 min at 3–3.5 ml/min under ketamine/xylazine anesthesia. Brains were extracted, post-fixed overnight in 4% PFA in PBS at 4°C, and coronally sectioned in PBS at 70 μm thickness with a vibratome. Per brain, a similar set of three sections was selected for immunostaining: an anterior section at the level of the caudate/putamen and anterior forceps of the corpus callosum, a middle section at 0.7 mm posterior to the anterior section, and a posterior section at 0.7 mm posterior to the middle section.

Brain sections were permeabilized with 0.5% Triton X-100 in PBS for 15 min. For immunostaining against VIP, permeabilization was followed by antigen retrieval in 10 mM trisodium citrate pH 6 with 0.05% Tween 20 at 80°C for 15 min at 300 rpm in an Eppendorf ThermoMixer. To block non-specific antibody binding, sections were incubated in 0.2% bovine serum albumin (BSA), 5% normal donkey serum (NDS), 0.2% glycine, 0.2% L-lysine hydrochloride, and 0.02% NaN_3_ in PBS for 1 h. Primary antibodies were applied overnight or up to 72 h in 0.3% Triton X-100, 2% BSA, and 0.02% NaN_3_ in PBS at 4°C with gentle shaking. The following primary antibodies were used: rabbit anti-PV (Abcam, ab11427, 1:500), rabbit anti-SST (Peninsula Laboratories, T4103.0050, 1:500), rabbit anti-VIP (ImmunoStar, 20077, 1:400), and mouse anti-mHTT (EM48; Millipore, MAB5374, 1:500). After washing three times 10 min with PBS, sections were incubated with Alexa Fluor 488 and/or Cyanine Cy3-conjugated secondary antibodies derived from donkey (Jackson ImmunoResearch Laboratories, 1:250) and NeuroTrace 640/660 (Invitrogen, N21483, 1:500) in 0.3% Triton X-100, 3% NDS, and 0.02% NaN_3_ in PBS with gentle shaking for 1 h at room temperature. Sections were washed in PBS for 10 min, followed by nuclear counterstaining with 0.5 μg/ml DAPI in PBS for 10 min. After washing in PBS for 10 min, sections were mounted with ProLong Glass Antifade Mountant (Invitrogen). Images were acquired with a Leica TCS SP8 confocal microscope.

Image processing and/or analysis was performed with Fiji (Schindelin et al., 2012). The boundaries of the primary motor cortex and cingulate cortex were defined by anatomical landmarks according to the Allen Reference Atlas — Adult Mouse Brain, atlas.brain-map.org. The cortical layers were identified by cytoarchitecture based on DAPI and NeuroTrace staining (see Supplementary Figures 1A,D). The Fiji Cell Counter plugin was used for cell counting of immunopositive interneurons and for colocalization analysis. tdTomato+ and NeuroTrace+ cells were counted using CellProfiler 3.0.0 (McQuin et al., 2018). Quantification of tdTomato+ and PV+ terminals was done as previously described (Burgold et al., 2019). Aggregate area was quantified using a custom-written macro in Fiji. Image analysis was performed blindly.

### Statistical analysis

GraphPad Prism 9.2.0 (GraphPad Software) was used for graphical representation and statistical analysis. Data are expressed as mean ± SD. Differences were considered statistically significant with *p* < 0.05.

## Data availability statement

The original contributions presented in this study are included in the article/Supplementary material, further inquiries can be directed to the corresponding author.

## Ethics statement

The animal study was reviewed and approved by the Government of Upper Bavaria.

## Author contributions

KV performed the experiments, analyzed the data, and designed the figures. EKS-T performed immunostainings in R6/2 mice at the initial stages of the project. RK and ID supervised the project. ID conceived the project and wrote the manuscript with contribution from KV. All authors contributed to the article and approved the submitted version.
