# Supplementary figures and images for: Distinct histological alterations of cortical interneuron types in mouse models of Huntington’s disease
Source: Front Neurosci. 2022 Sep 26;16:1022251. doi: 10.3389/fnins.2022.1022251 (PMC9549412; doi:10.3389/fnins.2022.1022251)

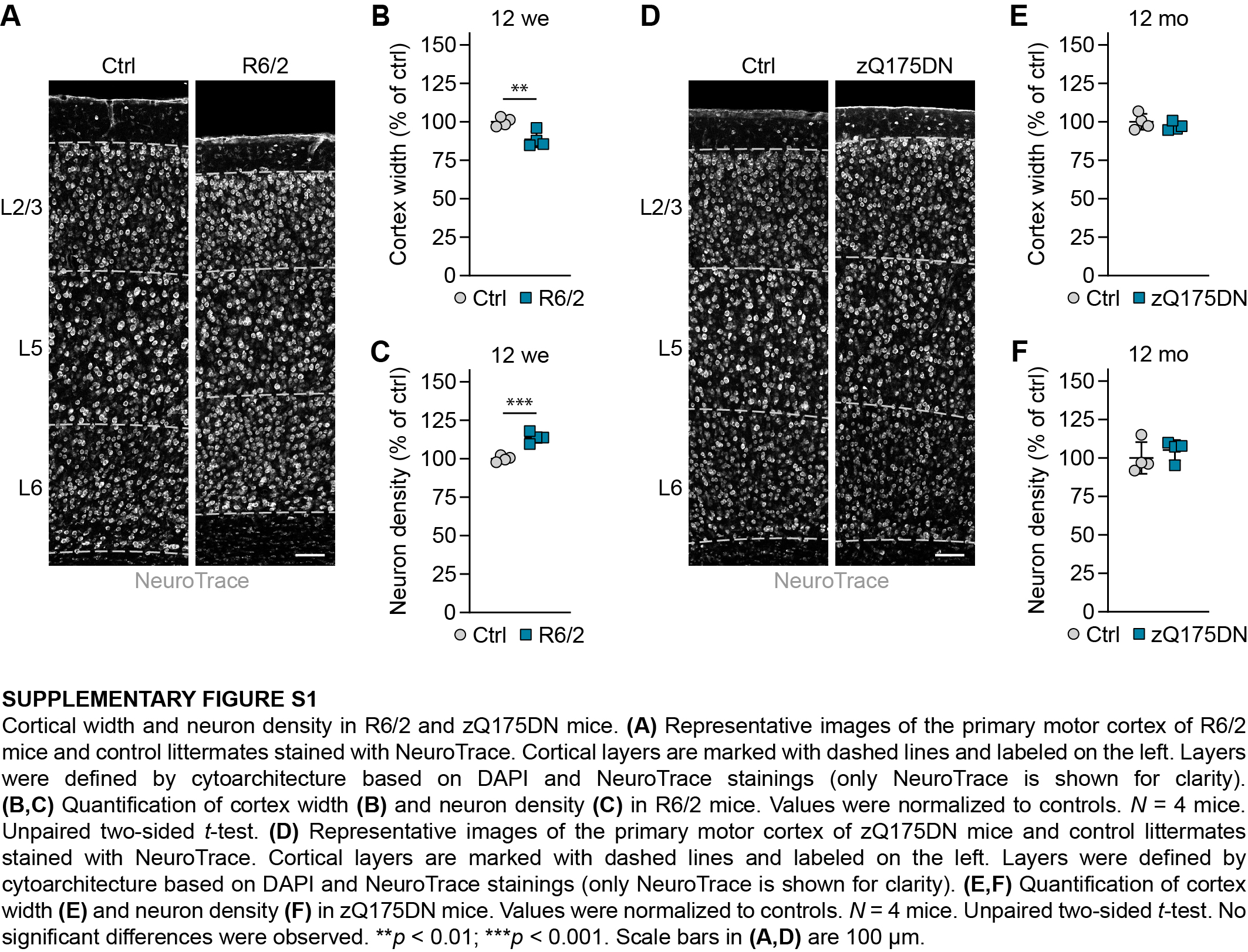

Supplement: Supplementary file 1 [file Image_1.jpg]
